# How Happy Do These Animals Look? Exploring Factors Influencing Children’s Perceptions of Animal Welfare at the Zoo

**DOI:** 10.3390/ani15111595

**Published:** 2025-05-29

**Authors:** María Ignacia Vera-Concha, Manuel Rojas, Daniel Cartes, Maria Camila Ceballos, Mari Carmen Villarroel, Martín Pérez, Vladimir Venegas, Cristóbal Briceño, Javiera Calderón-Amor, Daniela Luna

**Affiliations:** 1 Departamento de Ciencia Animal, Facultad de Ciencias Veterinarias y Pecuarias, Universidad de Chile, Santiago 8820808, Chile; maria.vera.concha@ug.uchile.cl; 2 Departamento de Ingeniería Industrial, Facultad de Ciencias Físicas y Matemáticas, Universidad de Chile, Santiago 8320198, Chile; manuelrojas@uchile.cl; 3 Departamento de Ciencias Clínicas, Facultad de Ciencias Veterinarias y Pecuarias, Universidad de Chile, Santiago 8820808, Chile; nncartes@uchile.cl; 4 Faculty of Veterinary Medicine, University of Calgary, 2500 University Drive NW, Calgary, AB T2N 1N4, Canada; mariacamila.ceballos@ucalgary.ca; 5 Escuela de Pregrado, Facultad de Ciencias Veterinarias y Pecuarias, Universidad de Chile, Santiago 8820808, Chile; mari.villarroel@uchile.cl (M.C.V.); martin.perez.s@uchile.cl (M.P.); vnvnege@uchile.cl (V.V.); 6 Departamento de Medicina Preventiva, Facultad de Ciencias Veterinarias y Pecuarias, Universidad de Chile, Santiago 8820808, Chile; cristobal.briceno@uchile.cl; 7 Escuela de Graduados, Facultad de Ciencias Veterinarias, Universidad Austral de Chile, Valdivia 5090000, Chile

**Keywords:** animal welfare, animal needs, children and adolescents’ perception, zoo animals, qualitative assessment

## Abstract

Understanding how children and adolescents perceive zoo animal welfare provides insights into public views on captive conditions. This study used a mixed-methods approach to investigate these perceptions. A total of 254 participants (aged 7–18) were surveyed, with 113 completing the full circuit, which included species from distinct phylogenetic groups: monkey, macaw, caiman, frog, fish, and tarantula. Qualitative analysis (*n* = 254) evaluated children’s recognition of animal needs, whereas quantitative analysis (*n* = 113) examined how phylogenetic distance, participants’ emotional state, and overall welfare perception influenced animal and environmental assessments—a structured survey evaluating perceived animal health, enclosure conditions, and behavioral expression. Participants’ understanding of animal welfare extended beyond basic needs, recognizing the importance of species-specific behaviors and appropriate environments. Environmental and animal assessment scores were significantly influenced by species, participants’ emotional states, and overall welfare perceptions: fish received the highest scores, positive emotions were associated with higher ratings, and a better overall welfare perception correlated with more favorable assessments. We inferred that phylogenetic distance, emotional state, and general welfare impressions shaped how children and adolescents evaluate animal welfare. The study supported zoo-based educational strategies and reinforced the role of zoos in promoting welfare awareness and conservation-oriented attitudes.

## Simple Summary

Understanding how children and adolescents perceive animal welfare in zoos is important for improving both animal care and educational programs. This study examined how 254 children and adolescents aged 7 to 18 assessed six animals in their enclosures at a zoo: a monkey, a parrot, a caiman, a frog, a fish, and a tarantula. Participants were asked about the animals’ needs (*n* = 254) and how they felt emotionally, how they perceived each animal’s welfare, and how they rated the animals’ enclosures (*n* = 113). Participants understood animal welfare as more than just providing food or water; they also recognized the importance of natural behaviors and proper environments for each species. Participants’ ratings were influenced by their emotional state, their overall impression of each animal’s welfare, and the evolutionary closeness of the species observed. Interestingly, fish received the highest environmental and animal ratings, even higher than mammals and birds. Participants who reported positive emotions gave more favorable assessments. We concluded that emotions and individual perceptions play key roles in how children and adolescents assess animal welfare, providing valuable insights for zoo educational efforts.

## 1. Introduction

Historically, zoos were primarily regarded as places of entertainment. However, in recent decades, they have undergone substantial functional and structural transformations, evolving into modern institutions emphasizing conservation, education, and research alongside recreation [1]. Besides these changes, public perceptions and expectations have shifted [2]. Although modern zoos are recognized for their educational and conservation efforts [3], concerns about inadequate housing conditions and the welfare of captive animals persist [1].

Recently, the perception of zoo animal welfare has become a growing area of interest, driven by societal expectations and institutional efforts to improve the treatment and housing of animals in human care [4]. This topic has been explored mainly through a social sciences approach [5], with limited integration of an animal welfare science framework [6]. One of the most widely used frameworks, the Five Domains Model, provides a comprehensive approach to assessing animal welfare. It includes four physical domains—nutrition, physical health, environment, and behavioral interactions—which collectively influence the fifth domain: affective states [7]. Importantly, since its 2020 update, domain 4 (behavioral interactions) explicitly incorporates interactions with humans, conspecifics, and the environment, making it particularly relevant to studies examining human–animal relationships such as the present one.

Phillips et al. [6] identified three main categories that influence visitors’ perceptions of animal welfare: factors related to the visitors themselves (e.g., gender), the environment (e.g., enclosure design), and the animals (e.g., visible behavior or appearance). Among visitor-related factors, gender [8] and emotional states [5] have been linked to variations in how the welfare of the exhibited/observed animals is perceived. For instance, Reade and Waran [8] reported that female respondents were significantly more likely to perceive zoo animals as bored than males. Moreover, Miller et al. [9] demonstrated that visitors’ positive emotional experiences, such as a sense of awe or wonder, were correlated with favorable animal welfare assessments. Environmental factors, including enclosure naturalness, space, and maintenance, have also been associated with positive perceptions of animal welfare [9,10]. Regarding animal-related factors, most research has focused on mammals and birds [5,9,11], with little to no attention given to invertebrates [6]. Among the factors examined/reported within the studied taxa, animal behavior has been identified as an aspect influencing visitors’ perceptions. For example, active behaviors in jaguars were linked to more favorable assessments of welfare and naturalness by visitors [12]. Conversely, in a study involving gorillas, low activity levels were interpreted by visitors as signs of unhappiness, even though they were considered typical by experts [5].

However, these findings are largely based on adult samples, and little is known about how children perceive and respond to animal-related factors in zoo settings. Regarding children, research has primarily focused on constructs such as attitudes [13,14], empathy [15], opinions [16], and knowledge [17], often in the context of domestic animals [17]. Studies specifically addressing children’s perception of animal welfare remain limited [18,19,20]. Nonetheless, based on existing literature, children can interpret and critically evaluate the welfare of observed animals [19]. Rocha et al. [21] reported that children are capable of identifying emotional expressions in animals observed in zoo settings, particularly if they have prior experience with animals or have been exposed to emotionally relevant events. Factors such as age, pet ownership, and visits to animal sanctuaries predicted children’s ability to recognize animal emotions, highlighting the importance of both developmental stage and environmental context in shaping children’s emotional sensitivity to captive animals. More recent studies have reinforced the importance of these factors. Burris [22] reported that intrinsic traits such as empathy and previous interactions with animals influenced how children engage with zoo animals and interpret their needs. Similarly, Phillips et al. [23] reported that participatory educational programs improved children’s conservation attitudes, suggesting that early interventions may foster more informed and empathetic perceptions of animal welfare.

Together, these studies highlighted the importance of studying children’s perception of animal welfare from an animal welfare science perspective. Understanding how children perceive welfare and factors influencing these perceptions is essential, not only for developing educational programs [5], encouraging public engagement, and increasing support for zoo conservation initiatives [24], but also for directly enhancing animal welfare awareness in society. Therefore, this study had two main objectives: first, to assess children’s and adolescents’ recognition of animal needs using a qualitative approach; and second, to examine how species’ phylogenetic distance, children’s and adolescents’ emotional state and overall welfare perception influence their environmental and animal assessment in a zoo setting.

## 2. Materials and Methods

### 2.1. Ethics Compliance

This research received ethical approval from the Investigation Ethics Committee of the Faculty of Social Sciences at the University of Chile (Approval N° 27-35/2023). Participation was voluntary, with informed consent obtained from all legal guardians and assent provided by the children and adolescents prior to completing the survey. Participants were informed that they could withdraw from the study at any point during data collection.

### 2.2. Study Site and Participants

The study was conducted at Buin Zoo, a private zoological institution located in the Metropolitan Region of Santiago, Chile. The zoo is regionally accredited by the Latin American Association of Zoos and Aquariums (ALPZA) and globally by the World Association of Zoos and Aquariums (WAZA).

Six enclosures, each representing a distinct phylogenetic group, were included in the study: “Monkey” (capuchin monkey, *Sapajus apella*, ~50 m^2^, seven individuals, grouped), “Macaw” (blue-and-yellow and scarlet macaws, *Ara ararauna*, *Ara macao*, ~70 m^2^, four individuals, grouped), “Caiman” (broad-snouted caiman, *Caiman latirostris*, ~40 m^2^, solitary), “Frog” (Chilean helmeted bullfrog, *Calyptocephalella gayi*, ~0.12 m^2^, solitary), “Fish” (cardinal tetra, *Paracheirodon axelrodi*, ~0.8 m^2^, ~50 individuals, grouped), and “Tarantula” (Indian ornamental tree spider, *Poecilotheria regalis*, ~0.09 m^2^, solitary). These species were selected to represent distinct phylogenetic groups (mammals, birds, reptiles, amphibians, fish, and invertebrates), ensuring taxonomic diversity. Additional selection criteria included enclosure visibility, accessibility within the zoo’s layout, and close distance between selected enclosures. Enclosure names are used for reference throughout the text.

We interviewed 254 children and adolescents (145 identified as female, 107 as male, and two as other or preferred not to disclose), ranging in age from 7 to 18 years (Mean = 11.26, Standard Deviation = 2.34), who visited the zoo accompanied by their legal guardians. Participants came from various regions of Chile and attended different types of schools: public (state-funded public schools, *n* = 74), subsidized (private schools that receive partial government funding, *n* = 98), private (*n* = 61), homeschool (*n* = 1), or not reported (*n* = 20). For the qualitative analysis, responses from all 254 children and adolescents were used. However, only 113 participants completed the full six-animal circuit. To ensure consistent comparisons across species, only these complete responses were included in the descriptive and quantitative statistical analysis. Participants were selected based on a minimum required reading comprehension level and basic knowledge of living organisms, as outlined in the first- and second-grade curricula of the Chilean Ministry of Education [25].

### 2.3. Data Collection Instrument

A structured survey was developed to assess participants’ recognition of animal needs, their emotional state while observing animals, and their perception of animal welfare (Supplementary File S1). The instrument was based on previous research on zoo visitors’ perceptions and emotional responses [5,9,26], studies on children’s perceptions [19], and the Five Domains Model of animal welfare [7]. It was created in Spanish and refined with input from a Spanish teacher and a sociologist from the Faculty of Veterinary and Livestock Sciences at the University of Chile.

The survey was administered while participants were actively observing the animals and consisted of two main sections. The first section explored participants’ recognition of animal needs through an open-ended question; the second included a self-reported emotional response and a series of Likert-type questions assessing perceptions of animal welfare.

The first section comprised an open-ended question in which participants were asked: “What do these animals need to be well and happy?” Responses were analyzed qualitatively to assess participants’ conceptualization of animal needs.

The second section included three subsections. The first subsection assessed participants’ emotional state while observing the animals, using the question: “What emotion did you feel most while watching these animals?” Participants selected one of eight illustrated emotions: neutral, disgust, fear, sadness, curiosity, peace, happiness, and admiration.

The second subsection assessed overall welfare perception using a single-item question: “How do you consider the welfare status of these animals?” representing a global judgment of welfare based on a single response. Responses were recorded on a five-point Likert-type scale, illustrated with facial expressions ranging from “very bad” to “very good”.

The final subsection, environmental and animal assessment, consisted of nine Likert-type questions evaluating participants’ perceptions of the animals’ environment and apparent condition (Table 1). These questions were designed to reflect the Five Domains Model framework for assessing animal welfare, excluding the nutritional domain. Responses were recorded using the same five-point Likert-type scale as in the previous subsection. Scores from these nine items were summed to generate a total environmental and animal assessment score, ranging from 9 to 45 points. Unlike the overall welfare perception score, which provides a global judgment based on a single response, the environmental and animal assessment score offered a composite measure of participants’ perceptions across multiple aspects of the enclosure and the animals’ observed condition.

### 2.4. Pilot Testing and Instrument Reliability

A pilot study was conducted one week prior to the main data collection with 20 children and adolescents visiting the zoo, following the methodologies outlined by Almeida and Sumozas [19]. The aim was to test the instrument’s reliability, evaluate comprehension, ensure the appropriateness of terms, and estimate the time required to complete the survey. Completion time was approximately 15 min per participant across the full circuit. No modifications were deemed necessary following the pilot. Instrument reliability was assessed using Cronbach’s alpha, yielding a coefficient of α = 0.847, which indicated high internal consistency among the environmental and animal assessment items.

### 2.5. Procedure

Data collection occurred from July to September 2023 during the zoo’s opening hours. Five trained research assistants conducted the surveys, accompanying participants along a predetermined route. The assessment began at the monkey enclosure, followed by the macaws, caiman, frog, and tarantula, and concluded at the fish exhibit. A systematic on-site sampling method, known as next-across-the-line, was used to recruit participants. An imaginary line was placed approximately 2 m from each enclosure, and every second group of visitors with children or adolescents crossing that line was invited to participate [10]. This method helped reduce selection bias by distributing participant recruitment across different times of the day. In groups with multiple eligible individuals, the second child from left to right was selected to randomize individual participation.

Once informed consent and assent were obtained, each participant received a printed version of the survey. Research assistants read the questions aloud while audio-recording the session for transcription. Participants responded verbally, although for some questions, such as those involving emotional state or welfare assessments, answers were occasionally indicated non-verbally by pointing at the response options on the sheet.

The survey included a section for each of the six selected enclosures. After completing the first section, participants could choose whether to continue. If participants or their guardians opted not to proceed, data collection ended at that point. No additional information about the animals was provided (to avoid influencing participants’ responses).

### 2.6. Data Analysis

Descriptive statistics, including means, standard deviations, and percentages, were used to summarize participants’ responses. All data were analyzed using the statistical program R (version 4.3.0; R Foundation for Statistical Computing, Vienna, Austria) [27], with statistical significance set at *p* < 0.05. For qualitative analysis, data from all 254 participants were included. In contrast, quantitative analysis was based only on the subset of participants that completed the entire circuit (*n* = 113).

To assess participants’ recognition of animal needs, Text mining analysis was applied to the transcribed responses for the open-ended question “What do these animals need to be well and happy?” The analysis conducted with the Tm 0.7–16 package [28] included text cleaning and preprocessing techniques, such as lowercase, number removal, punctuation, stopwords, and tokenization. In addition, a document matrix and a semantic corpus were utilized. Finally, n-grams and cluster analyses were done. Cluster analysis was performed using the cluster 2.1.8.1 package [29] and the skmeans 0.2–18 package [30] to group similar responses and identify patterns in participants’ recognition of animal needs. The optimal number of clusters was determined using the Calinski-Harabasz index [31]. Responses for each species were organized into clusters using the spherical k-means method.

A linear mixed-effects model was performed to examine the relationship between the total score of the environmental and animal assessment (nine Likert-type questions, ranging from 9 to 45 points) and the explanatory variables: phylogenetic closeness, self-reported emotional valence, and overall welfare perception.

For the variable phylogenetic closeness, a nominal variable was created to represent the phylogenetic closeness of the six species to humans, based on a phylogenetic tree created using phyloT v2.2023 [32]. From closest to more distant, the order was as follows: (1) monkey, (2) macaws, (3) caiman, (4) frog, (5) fish, and (6) tarantula.

Self-reported emotions in response to the question “What is the emotion that you felt the most, while observing these animals?” were categorized according to their valence, classifying them as positive (admiration, joy, curiosity, peace), negative (disgust, fear, sadness), and neutral (neutral).

Responses to the question “How do you consider the welfare status of these animals?” were scored on a five-point Likert scale. Due to low frequency of some responses, scores 1 (“very bad”), 2 (“bad”), and 3 (“regular”) were combined into a single category, whereas scores 4 (“good”) and 5 (“very good”) were retained as individual categories.

The linear mixed-effects model was conducted using the lmer function from the Lme4 1.1-37 package [33], with explanatory variables entered as fixed effects and participants included as a random effect. Model assumptions were visually assessed using residual plots. Post hoc comparisons were conducted using the Tukey test with the emmeans 1.10.4 package [34]. The following model was used:

Environmental and animal assessment score ~ Phylogenetic closeness + Emotional valence + Overall welfare perception + (1|child)

## 3. Results

### 3.1. Children’s and Adolescents’ Recognition of Animal Needs

Children’s descriptions of what each animal needed were grouped in four clusters for each species (Table 2). Each cluster represented a set of conceptually related terms that reflect patterns in participants’ recognition of animal needs. The cohesion of each cluster, measured through mean silhouette coefficients, ranged from 0.0974 to 0.2092. Across all species, clusters consistently included references to environmental (e.g., “space”, “habitat”, “environment”) and nutritional needs (e.g., “eating”, “water”), suggesting that these are central concepts in children’s and adolescents’ understanding of animal welfare.

The monkey cluster (silhouette = 0.0974) included references to nutrition (e.g., “food”), opportunities for movement and play, resting areas, and cleanliness. In the macaw cluster (0.1793), common themes were water access, space for flying, social interaction, and appropriate perching. The caiman cluster had the highest cohesion (0.2092), with responses focusing on aquatic environments, space, and comfort. For the frog (0.1693), children mentioned needs related to moisture, vegetation, light, space, and food. The fish cluster (0.1800) included water quality, swimming space, hiding places, and diet. Descriptions for the tarantula (0.1668) emphasized hiding spots, substrate, food availability, and hygiene.

### 3.2. Environmental and Animal Assessment

**Figure 1:**
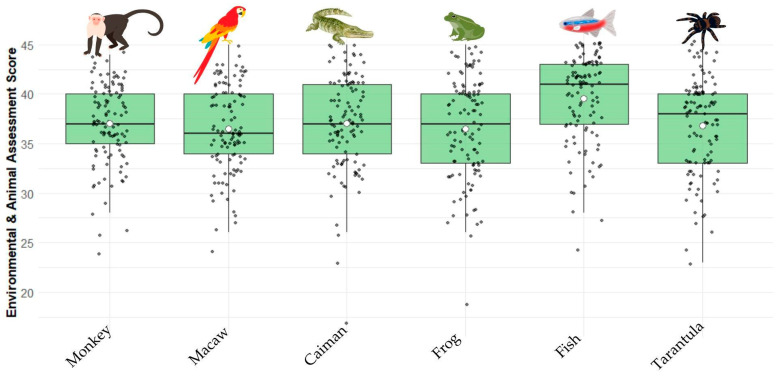
Boxplots showing the distribution of environmental and animal assessment scores (range: 9–45) assigned to each species by children and adolescents (*n* = 113). Each black dot represents an individual participant’s score. White circle indicate the mean, and the horizontal line inside each box represents the median. The vertical spread reflects variability in responses; points outside the whiskers represent potential outliers.

**Figure 2:**
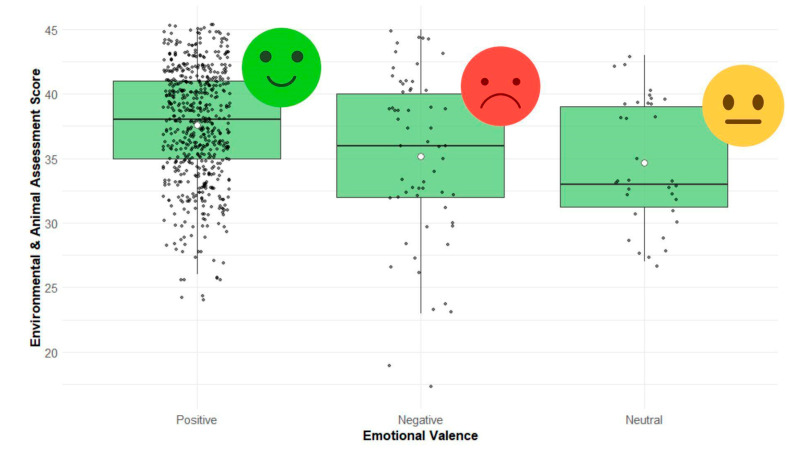
Boxplots showing the distribution of environmental and animal assessment scores (range: 9–45) based on self-reported emotional valence while observing zoo animals (*n* = 113). Black dots represent individual responses, white circles indicate the mean, and the horizontal line inside each box represents the median. Emotional valence categories were positive (e.g., happiness, curiosity, admiration), neutral, and negative (e.g., fear, sadness, disgust). Points beyond the whiskers represent potential outliers.

Out of the 113 participating children and adolescents, the average score on the environmental and animal assessment survey was 37.23 ± 4.70 (Figure 1). Across all species, most of the emotional responses were positive (82.3%), with fewer negative (8.7%) and neutral (5.0%) responses (Figure 2). The tarantula elicited the highest proportion of negative emotions (30.9% of participants), whereas the fish received the most positive responses (99.1% of all emotional responses, with only one child reporting a negative response). Overall welfare perceptions were predominantly favorable, with most responses rated as “good” (score 4, 57.4%) or “very good” (score 5, 33.8%). Only 8.8% of the assessments fell into the “regular”, “bad”, or “very bad” categories.

Environmental and animal assessment scores were significantly associated with phylogenetic closeness (F _(5, 548)_ = 19.56, *p* < 0.0001), self-reported emotion valence (F _(3, 605)_ = 12.33, *p* < 0.0001), and overall welfare perception (F _(2, 581)_ = 38.16, *p* < 0.0001).

In post hoc comparisons, fish received significantly higher environmental and animal assessment scores than any other species evaluated: monkey (t = −7.14, *p* < 0.0001), macaw (t = −8.59, *p* < 0.0001), caiman (t = −6.63, *p* < 0.0001), frog (t = −7.80, *p* < 0.0001), and tarantula (t = −5.85, *p* < 0.0001).

Participants gave higher environmental and animal assessment scores when rating an animal’s overall welfare as “Very good” (5 points), compared to “Good” (4 points; t = −6.46, *p* < 0.0001) or any lower rating (pooled “Regular”, “Bad”, or “Very bad”; t = −8.33, *p* < 0.0001). Likewise, “Good” ratings were associated with higher scores than the pooled lower ratings (t = −5.00, *p* < 0.0001). Participants who reported positive emotions assigned higher environmental and animal assessment scores than those reporting neutral (t = 3.50, *p* = 0.001) or negative emotions (t = 4.00, *p* = 0.0001), with no significant difference between neutral and negative responses (*p* = 0.99).

## 4. Discussion

Participants’ responses reflected a structured yet species-dependent understanding of animal welfare. Clusters generated through k-means semantic analysis revealed consistent references to physical environment, nutrition, and health-related needs across taxa. However, in the case of more socially or cognitively salient animals (e.g., primates and parrots), responses also included references to behavioral and mental domains, such as companionship, play, and emotional states. This partially aligns with the Five Domain Model [7], suggesting that while children recognize basic biological needs across species, their awareness of affective or cognitive needs emerges more clearly for animals perceived as emotionally relatable. This pattern was consistent with previous studies on children’s perceptions of pet needs [35], farm animal welfare [17], and wildlife welfare [18], which also reported a greater emphasis on biological functioning needs over affective states.

Tinbergen’s framework of proximate and ultimate needs [36] provides a useful lens to interpret the semantic clustering results. Basic, proximate needs—e.g., food, water, and shelter—were mentioned across all species. However, only phylogenetically closer animals, like the monkey and the macaw, were associated with more complex or ultimate needs, including play, flight, companionship, and social interaction—needs that support emotional expression, bonding, and cognitive stimulation. In contrast, responses for more evolutionarily distant species (frog, tarantula, and fish) focused mainly on survival essentials. This pattern suggests that evolutionary proximity may influence how children and adolescents perceive what animals need to be well, with closer species prompting more detailed and emotionally nuanced interpretations [37,38]. These perceptions may be shaped not only by recognition of species-specific behaviors but also by empathetic engagement and perceived similarity to humans. Supporting this, terms such as play, friend, companionship, and stress appeared more frequently for primates and parrots, aligning with previous research that suggests empathy and moral concern increased with morphological or evolutionary closeness [39,40].

This interpretation was further supported by the semantic clustering results, which revealed how children and adolescents assigned various types of needs to each species with varying degrees of consensus. Higher silhouette values, e.g., that of the caiman, point to more consensus and shared perceptions, whereas lower values, e.g., that of the monkey, indicate greater variability in responses—possibly reflecting less agreement among participants and more diverse interpretations of what that animal might need. This variation may stem from the perception of certain species as emotionally or cognitively complex, prompting broader and more individualized attributions.

Phylogenetic closeness, emotional valence, and overall welfare perception were significantly associated with environmental and animal assessment scores. Among the species, in post hoc results, fish was rated significantly higher than all others. This suggests that phylogenetic closeness alone does not predict better-perceived welfare. In fact, more distantly related species were rated more positively than mammals, birds, or reptiles. This pattern aligns with Prokop et al. [41] who reported that conservation attitudes are shaped more by observable traits, such as posture or gaze, than by evolutionary relatedness. Additional factors may have influenced perceptions in this study: the fish’s small size, naturalistic setting, and high group density may have contributed to its favorable assessment, despite not being explicitly measured [10,42,43].

Another interpretation is that participants perceived the fish’s needs as simpler and easier to satisfy, which may have led to more favorable welfare assessments. In the case of the fish, qualitative responses focused on basic elements—food, water, and space—with little mention of emotional or behavioral needs. In contrast, animals such as macaws and monkeys were associated with more complex expectations, including play, freedom, and companionship. We inferred that children may apply higher welfare standards to animals perceived as more cognitively or emotionally complex, resulting in more critical assessments if those expectations appear unmet. Animals regarded as emotionally distant or less expressive may benefit from more favorable judgments based on simpler perceived needs. This perspective echoes Harrison and Hall [44], who reported that perceived human-animal similarity increased the attribution of emotional and communicative traits—factors that could intensify empathetic concern and, in turn, lead to stricter welfare assessments.

Order effects may also have influenced the results. Although phylogenetic distance and the order in which animals were observed were analyzed separately, they overlapped in this study, with the fish being the last animal presented during the visit. Its high rating could reflect increased familiarity with the setting, reduced observer stress, or heightened empathy over time. However, this contrasts with Davey [45], who reported that animals observed earlier in zoo visits tended to receive more favorable evaluations. The fact that the fish scored highest, despite being observed last, implies that species traits or perceived needs played a more decisive role than order alone.

Emotional valence was also associated with environmental and animal assessment scores. Participants who reported positive emotions during the observation assigned higher environmental and animal scores than those who reported neutral or negative emotions. This result is consistent with previous studies on adult zoo visitors, where positive emotions were linked to more favorable welfare perceptions [9,26]. Although prior research largely focused on positive emotions, this study contributes by incorporating a broader range of emotional responses.

Importantly, emotional valence remained a significant predictor of environmental and animal assessment scores, even after controlling for phylogenetic closeness and overall welfare perception, implying that participants’ emotional state independently influenced their assessments. However, because responses were strongly skewed toward positive emotions, it was not possible to fully explore how emotional valence varied by species. Even so, descriptive data offered some insights. The fish, which received the highest environmental and animal assessment scores, also elicited overwhelmingly positive emotions (99.1%), with only one participant reporting a negative response. This convergence may reflect the species’ small size, low perceived threat, and calming, visually appealing exhibit—factors that could have enhanced the emotional experience and, in turn, led to more favorable assessments. This aligns with Luebke et al. [26], who reported that observing active animals elicited more positive emotions in zoo visitors. In our study, fish were constantly in motion, which may have contributed to both emotional assessments and environmental and animal assessments. Similarly, Sherman et al. [10] reported that increasing giraffe group size improved welfare ratings without affecting visitor emotions, suggesting that emotional responses may depend more on visible animal behavior than on actual welfare conditions alone.

Based on the association between overall welfare perception and the environmental and animal assessment scores, we inferred that the participants’ general impressions of animal welfare closely align with their more detailed assessments. Although the overall welfare perception reflects a single, intuitive judgment, the environmental and animal assessment score encompasses multiple dimensions such as enclosure space, naturalness, animal comfort, and perceived happiness. Furthermore, the coherence between these two measures indicates that participants may integrate specific environmental and animal cues into their broader welfare perceptions. This interpretation was further supported by qualitative data, where participants spontaneously referred to similar themes, such as space, habitat, water, and species-specific needs, when describing animals’ needs. This result supports the validity of using a multi-item and mixed methods approach to assess how children and adolescents perceive animal welfare in zoo settings. Future studies could examine whether certain domains within the assessment (e.g., physical environment or behavioral interactions) weigh more heavily in shaping overall impressions or whether discrepancies emerge when emotional responses are particularly strong.

This study provides valuable insights into how children and adolescents perceive animal welfare in zoo settings, offering a foundation for developing more targeted educational strategies. Participants’ responses often reflected observable aspects of the enclosures, suggesting that exhibit design plays a key role in shaping welfare perceptions. These results highlight the importance of integrating educational content into enclosure design [4] and environmental enrichment programs [5,9] to enhance public understanding and engagement. Promoting positive welfare perceptions is also essential for strengthening the public image of modern zoos, which can in turn increase visitation and support their educational and conservation goals [1,43]. Moreover, educational strategies in zoos also serve as critical tools for promoting captive breeding and biodiversity conservation programs, particularly for threatened species, by fostering behavioral change and public support [46]. These findings also point to potential future directions. Longitudinal research could examine how early exposure to zoo-based welfare education shapes conservation attitudes, ethical reasoning, and pro-animal behaviors over time. In addition, the development of participatory tools (e.g., interactive digital surveys) could allow children to reflect on their welfare perceptions while also generating valuable data for researchers and educators.

Beyond enclosure features, our results emphasize that welfare judgments are shaped by a combination of factors, including species characteristics and participants’ emotional responses toward the animal. This highlights the need for interdisciplinary strategies in zoo education that not only convey biological information but also actively engage visitors emotionally. Designing educational interventions that combine knowledge from animal behavior, psychology, and science communication can help create experiences that are both evidence-based and emotionally meaningful. Future research should further explore how variables such as species familiarity [43], cultural context [47], observed animal behavior [9,12], and individual visitor differences, e.g., gender [8], influence the way animal welfare is perceived.

This study had some limitations. The sample was restricted to children and adolescents visiting a single zoo in Chile, limiting the generalizability of the results—particularly to individuals from various socioeconomic backgrounds who may have less access to such institutions. Cultural and educational factors likely influenced participants’ perceptions, as exposure to animals and conservation knowledge can vary widely [47]. Social desirability bias may also have played a role, as some participants who voiced critical opinions still gave high scores on Likert-scale items, possibly influenced by the positive zoo environment. Although mixed methods were used to reduce bias, open-ended questions focused on animal needs rather than welfare conditions. Future studies should include questions that specifically address perceived welfare to gain a more complete picture of children’s and adolescents’ assessments.

## 5. Conclusions

This study contributes to understanding the factors influencing children’s and adolescents’ perceptions of animal welfare and needs in zoological settings. Qualitative data revealed that children and adolescents recognized a range of animal needs—particularly environmental and nutritional—and demonstrated the ability to differentiate these needs across species. Quantitative findings showed that environmental and animal assessments varied by observed species and were influenced by the emotional responses elicited, although not directly linked to phylogenetic closeness.

These results highlight the potential of educational strategies to shape how younger audiences understand and evaluate animal welfare. Promoting early awareness of animal needs may not only foster more informed and empathetic zoo visitors but also contribute to long-term cultural shifts that prioritize animal welfare. This has practical implications for the design of zoo-based educational programs and suggests the value of integrating animal welfare education into formal school curricula.

In turn, these efforts may promote more critical and constructive public engagement with captive animal care, contributing to improved welfare standards. Despite the study’s limitations, it provides a foundation for future research and interventions aiming to enhance both educational outcomes and the lived experiences of animals in human care.

## Figures and Tables

**Table 1:** Environmental and animal assessment questions and their corresponding animal welfare domains.

Question	Domain
How healthy do these animals look?	Health
How clean do you think the animal’s environment is?	Physical Environment
How spacious do you think the place is for these animals?	Physical Environment
How similar is this place to their natural environment?	Physical Environment
How appropriate do you consider their resting places?	Physical Environment
How entertaining do you find their environment?	Behavioral Interactions
How comfortable do they look in their environment?	Behavioral Interactions
How appropriate do you find the number of animals within the space?	Behavioral Interactions
How happy do you find these animals?	Mental State

**Table 2:** Clustering results of k-means cosine algorithm of children’s and adolescents’ responses (organized by species) and most representative words per cluster.

Cluster	Species					
	Monkey	Macaw	Caiman	Frog	Fish	Tarantula
1	food	free	habitat	space	food	**soil**
	habitat	**air**	natural	food	water	plants
	water	**macaw**	food	**feed**	habitat	**hide**
	live	company	**similar**	large	**fishes**	**log**
	clean	friend	comfortable	natural	environment	**insects**
2	space	**fly**	water	**food**	plants	space
	food	space	food	habitat	**algae**	rest
	free	**achieve**	plants	comfortable	**rocks**	live
	large	rest	**pond**	water	water	clean
	play	branches	**lie-down**	**healthy**	rest	love
3	eat	habitat	company	plants	space	food
	**banana**	natural	care	water	large	environment
	**take**	food	play	**quiet**	swim	habitat
	**climb**	need	friend	**sea**	food	large
	play	companionship	**warm**	love	**relation**	**sleep**
4	free	food	space	**humidity**	**ocean**	comfortable
	**tree**	water	swim	environment	**wide**	care
	home	space	**heat**	**leaves**	**quantity**	eat
	**jungle**	large	water	need	**house**	branches
	**stress**	home	**open**	**light**	**shelter**	**area**
Silhouette metric	0.0974	0.1793	0.2092	0.1693	0.1800	0.1668

Children’s and adolescents’ responses were grouped into clusters based on semantic similarity. The table presents five representative words per cluster, organized by species. Words in bold indicate terms that appeared only in the responses for that species and did not occur in other species’ clusters.

## Data Availability

The original data presented in this study are openly available on Zenodo at https://doi.org/10.5281/zenodo.15342854.

## Supplementary Materials

The following supporting information can be downloaded at https://www.mdpi.com/article/10.3390/ani15111595/s1, File S1: Full survey instrument used in the study, including all closed- and open-ended items.
