# Supplementary material for: How Happy Do These Animals Look? Exploring Factors Influencing Children’s Perceptions of Animal Welfare at the Zoo
Source: Animals (Basel). 2025 May 29;15(11):1595. doi: 10.3390/ani15111595 (PMC12153875; doi:10.3390/ani15111595)
Supplement: Supplementary file 1 [file animals-15-01595-s001.zip › animals-3656522-supplementary.pdf]

Supplementary File S1. Survey Instrument

SURVEY QUESTIONS

1. RECOGNITION OF ANIMAL NEEDS

1.1. What do these animals need to be well and happy?

____

2. CHILDREN'S EMOTIONAL STATE AND ANIMAL WELFARE ASSESSMENT

2.1. What emotion did you feel the most while observing these animals?

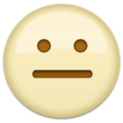	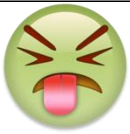	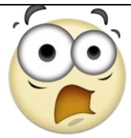	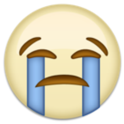	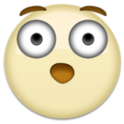	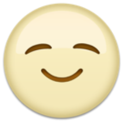	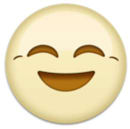	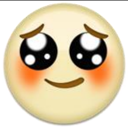
**Neutral**	**Disgust**	**Fear**	**Sadness**	**Curiosity**	**Peace**	**Happiness**	**Admiration**

2.2. How do you consider the welfare status of these animals?

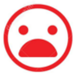	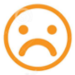		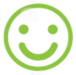	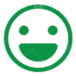
**Very bad**	**Bad**	**Regular**	**Good**	**Very good**

2.3. Environmental and animal assessment

Read each statement and point out the option that best matches your opinion	Very bad	Bad	Regular	Good	Very good
How healthy do these animals look?					
How healthy do these animals look?					
How spacious do you think the animal's environment is?					
How similar is this place to their natural environment?					
How appropriate do you consider their resting places?					
How entertaining do you find their environment?					
How comfortable do they look in their environment?					
How appropriate do you find the number of animals within the space?					
How happy do you find these animals?					

GENERAL INFORMATION

Enclosure: ________________ **Date and Time:** __/__/23 __:__

Children ID Nº: _______________

Gender: ☐ Female ☐ Male ☐ Prefer not to say ☐ Other: ____________

Age: _____ Years

School Type: ☐ Public ☐ Subsidized ☐ Private ☐ Homeschool

Which Region do you live in? ____________

Thank you for participating!
